# Supplementary material for: Intradermal injection of lidocaine with a microneedle device to provide rapid local anaesthesia for peripheral intravenous cannulation: A randomised open-label placebo-controlled clinical trial
Source: PLoS One. 2022 Jan 31;17(1):e0261641. doi: 10.1371/journal.pone.0261641 (PMC8803196; doi:10.1371/journal.pone.0261641)
Supplement: S1 Table — (DOCX) [file pone.0261641.s002.docx]

**S1 Table. The results of the linear mixed effects model of VAS score after the cannulation.**

| **Fixed effects** | | | | | |
| --- | --- | --- | --- | --- | --- |
| **Term** | **Estimate** | **SE** | **df** | **t-value** | **p-value** |
| Intercept | 3.59 | 1.01 | 180.65 | 3.55 | 0.0005 |
| Placebo | 37.89 | 1.49 | 129.76 | 25.32 | <0.0001 |
| No pretreatment | 36.12 | 1.52 | 131.31 | 23.76 | <0.0001 |
| **Random effects** | | | | | |
| **Term** | | **Variance** | | **SD** | |
| Intercept for subject | | 35.49 | | 5.96 | |
| Residual | | 69.51 | | 8.34 | |
