# Supplementary material for: Intradermal injection of lidocaine with a microneedle device to provide rapid local anaesthesia for peripheral intravenous cannulation: A randomised open-label placebo-controlled clinical trial
Source: PLoS One. 2022 Jan 31;17(1):e0261641. doi: 10.1371/journal.pone.0261641 (PMC8803196; doi:10.1371/journal.pone.0261641)
Supplement: S2 Table — (DOCX) [file pone.0261641.s003.docx]

**S2 Table. The results from the linear mixed effects model of skin numbness after the lidocaine injection.**

| **Fixed effects** | | | | | |
| --- | --- | --- | --- | --- | --- |
| **Term** | **Estimate** | **SE** | **df** | **t-value** | **p-value** |
| Intercept | 4.51 | 0.51 | 188.96 | 8.90 | <0.0001 |
| Distance = 2cm | 5.50 | 0.46 | 606.00 | 9.73 | <0.0001 |
| Distance = 3cm | 6.37 | 0.46 | 606.00 | 13.78 | <0.0001 |
| Time = 30 min | 6.48 | 0.59 | 266.76 | 10.96 | <0.0001 |
| Time = 45 min | 7.17 | 0.57 | 294.38 | 12.63 | <0.0001 |
| Distance = 2 cm × Time = 30 min | -3.08 | 0.65 | 606.00 | -4.71 | <0.0001 |
| Distance = 3 cm × Time = 30 min | -5.13 | 0.65 | 606.00 | -7.84 | <0.0001 |
| Distance = 2 cm × Time = 45 min | -3.92 | 0.65 | 606.00 | -6.00 | <0.0001 |
| Distance = 3 cm × Time = 45 min | -5.91 | 0.65 | 606.00 | -9.04 | <0.0001 |
| **Random effects** | | | | | |
| **Term** | **Variance** | **SD** | **Correlation** | | |
| Intercept for subject | 15.27 | 3.91 |  |  |  |
| t=30 min for subject | 13.85 | 3.72 | 0.20 |  |  |
| t=45 min for subject | 11.02 | 3.32 | -0.13 | 0.56 |  |
| Residual | 10.90 | 3.30 |  |  |  |
