# Supplementary material for: Intradermal injection of lidocaine with a microneedle device to provide rapid local anaesthesia for peripheral intravenous cannulation: A randomised open-label placebo-controlled clinical trial
Source: PLoS One. 2022 Jan 31;17(1):e0261641. doi: 10.1371/journal.pone.0261641 (PMC8803196; doi:10.1371/journal.pone.0261641)
Supplement: S1 File — (PDF) [file pone.0261641.s004.pdf]

Federal State Independent Educational Institution of Higher Education The First I. M. Sechenov Moscow State Medical University of the Ministry of Health of the Russian Federation (Sechenov University)

SYNOPSIS

of the clinical study titled:

Use of MicronJet600 hollow micro-needle injection system for lidocaine intradermal administration to ensure local anesthesia when inserting peripheral venous catheter

Principal investigator: Pavlov Chavdar Savov, Doctor of Medical Sciences, Professor, Head of the Scientific and Research Department of Innovation Therapy of the FGAOU VO [Federal State Independent Educational Institution of Higher Education] The First I. M. Sechenov Moscow State Medical University of the Ministry of Health of the Russian Federation (Sechenov University), Physician of the Hepatology Department of the Clinic for Internal Diseases Propedeutics, Gastroenterology, and Hepatology named after V. Kh. Vasilenko of UKB [University Clinical Hospital] No. 2.

Project coordinator: Aleksey Sergeevich Rzhevskiy, Junior Research Associate of the Biomedical Engineering Department at the FGAOU VO The First I. M. Sechenov Moscow State Medical University of the Ministry of Health of the Russian Federation (Sechenov University).

1. Contemporary and novel nature of the subject matter based on publications and patent documents

An ideal technique to ensure local anesthesia should be simple, effective, fast-acting, cost-effective, and cause minimum discomfort or pain to the subject [1]. Notwithstanding the vast number of methods currently available to provide local anesthesia, each method has its own limitations. Thus, standard subcutaneous injections are the most common way for administering local anesthetic agents, and allow for quick achievement of the desired result. However, subcutaneous needles may cause unwanted pain and stress in subjects, especially in subjects with the fear of sharp objects [2]. Alternatively, the available gels and creams based on local anesthetic agents ensure the pain-free achievement of anesthesia; however, the interval between their topical application and achieving an anesthetic effect constitutes approximately an hour, which significantly restricts their use [3]. In recent years, several minimum- and non-invasive methods of intradermal administration of local anesthetic agents have been developed, including: iontophoresis [4], ultrasound [5], and “jet injection” [6]. However, in view of complexity and the cost of the construction of required devices, these methods have not gained any special commercial popularity or common practical application.

Thus, in the planned study, we are going to study the efficacy of minimum invasive and pain-free intradermal administration of a local anesthesia agent, and as a result, local anesthesia followed by the insertion of peripheral venous catheter in the cubital vein, with the use of commercially available and registered in the Russian Federation (registration certificate, dated 23 May 2017, see the enclosed document) of MicronJet600 (NanoPass Technologies Ltd., Israel) hollow micro-needle injection system [7, 8]. This injection system has been developed for pain-free intradermal administration of medicinal products, with a particular focus on vaccination [8], In our study, we anticipate high efficacy of the use of this system to provide local anesthesia in intradermal administration of low doses of lidocaine 2% solution.

2. Purpose and objectives of the planned study

The primary objective of the planned study is to demonstrate the potential of pain-free and effective use of hollow microneedles to provide local anesthesia, as demonstrated by the use of intradermal administration of low doses of lidocaine with the use of MicronJet600 microneedle system.

Objectives of the study include the following:

a) Evaluate the efficacy of intradermal administration of low doses of lidocaine 2% solution with the use of MicronJet600, in order to reduce the pain syndrome associated with the peripheral venous catheter insertion procedure.

b) Identify potential side effects of intradermal administration of lidocaine with the use of MicronJet600.

c) Develop recommendations for the use of MicronJet600 in order to reduce the pain syndrome associated with the peripheral venous catheter insertion procedure.

3. Planned type of the clinical study

Prospective randomized cross-section placebo-controlled double-blind parallel-group clinical study

4. Object of the study and planned number of observed cases

Subjects of the clinic of propedeutics of internal diseases, gastroenterology, and hepatology named after V. Kh. Vasilenko of the UKB No. 2; with total of 80 subjects.

5. Specific methodology used in the planned study (+ method for statistical processing of results)

The pain experienced by subjects during insertion of 18G catheter in cubital vein will be assessed using the visual analog scale [9]. The obtained results will be statistically processed with the use of two-tailed t-test, with the confidence factor set at $p < 0.001$.

The clinical study design is presented in the form of the table:

	Group 1		Group 2	
	Left arm	Right arm	Left arm	Right arm
**Description of the peripheral venous catheter insertion**	A 18G catheter will be inserted in cubital vein immediately after the intradermal administration of 0.1 mL of 2% lidocaine solution in the administration site.	A 18G catheter will be inserted in cubital vein immediately after the intradermal administration of 0.1 mL of saline solution as placebo in the administration site.	A 18G catheter will be inserted in cubital vein immediately after the intradermal administration of 0.1 mL of 2% lidocaine solution in the administration site.	A 18G catheter will be inserted in cubital vein without the prior administration of lidocaine or saline solution.
**Number of subjects**	N=40		N=40	
**Performed assessments**	The pain experienced by subjects during the procedure will be assessed by them using the provided 100-point visual analog scale.			

	As an additional assessment, the administration site will be examined for any signs of swelling, edema or hematoma one hour after the use of MicronJet600 in all cases. Any complaints presented by subjects and directly related to this study will be recorded.<br>Also, in the case of lidocaine administration, the duration of loss of skin sensitivity (hereinafter referred to as DPPC) will be evaluated by means of a disposable small-gauge needle, caliber of 27 G and length of 13 mm, due to its point contact with the skin, i.e. insertion of the tip of the needle to a depth of 1-2 mm in the thickness of the skin at a right angle, at a distance of 1, 2 and 3 cm from the site of injection of lidocaine. An evaluation of DPPC will be performed 30, 60 and 90 minutes after the administration of lidocaine. When assessing the DDPC, a new disposable needle with a caliber of 27 G and a length of 13 mm will be used in each time interval. Quantitatively, the loss of skin sensitivity, that is, the pain experienced by subjects with the described contact of the surface of the skin with a needle caliber of 27 G and a length of 13 mm, will also be estimated with a 100-point visual analog scale.

Thus, in view of the fact that subjects will review the main concept of the study in their informed consent form (use of MicronJet600 in order to reduce the pain syndrome associated with the peripheral venous catheter insertion procedure), the placebo-controlled study with the first group of subjects will allow proving the lack of bias with regard to perception of MicronJet600 use, and the presence of an actual effect of reducing the pain syndrome associated with the peripheral venous catheter insertion following the intradermal administration of 2% lidocaine solution. On the other hand, the study involving the second group of subjects will allow assessing the degree of reducing the pain syndrome associated with the peripheral venous catheter insertion procedure following the intradermal administration of lidocaine using MicronJet600 versus the standard method for inserting peripheral venous catheter. During the research, in the case of each subject, manipulations will be carried out at the beginning with the right and then with the left hand.

The randomized distribution of subjects into two study groups will be made with the use of the sequential number method. Thus, each subject will be assigned a random number from the random number table. Then, the subjects allocated random even numbers will be assigned the first study group while the subjects allocated random odd numbers will be assigned the second study group.

All manipulations pertaining to this study will be performed in accordance with the Declaration of Helsinki of the World Medical Association, and Good Clinical Practice guidelines.

6. Expected study outcome

The practical efficacy of pre-administration anesthesia by way of intradermal injection of low doses of 2% lidocaine solution with the use of MicronJet600 injection system will be demonstrated along with the recommendations for successful completion of this procedure.

7. Research study sites

FGAOU VO The First I. M. Sechenov Moscow State Medical University of the Ministry of Health of the Russian Federation (Sechenov University), Clinic for Internal Diseases Propedeutics, Gastroenterology, and Hepatology named after V. Kh. Vasilenko of UKB [University Clinical Hospital] No. 2.

8. Organization support

For this study, MicronJet600 microneedle systems will be kindly supplied by NanoPass Technologies Ltd., Israel.

Principal Investigator: Ch. S. Pavlov

Project coordinator: A. S. Rzhevskiy

9. References

1. Houck, C.S. and N.F. Sethna, *Transdermal analgesia with local anesthetics in children: review, update and future directions.* Expert review of neurotherapeutics, 2005. **5**(5): p. 625-634.
2. Thurgate, C. and S. Heppell, *Needle phobia–changing venepuncture practice in ambulatory care.* Paediatric nursing, 2005. **17**(9): p. 15-18.
3. Zempsky, W.T., *Pharmacologic approaches for reducing venous access pain in children.* Pediatrics, 2008. **122**(Supplement 3): p. S140-S153.
4. Zempsky, W.T., et al., *Evaluation of a low-dose lidocaine Iontophoresis system for topical anesthesia in adults and children: A randomized, controlled trial.* Clinical therapeutics, 2004. **26**(7): p. 1110-1119.
5. O'Donnell, B.D. and G. Iohom, *An estimation of the minimum effective anesthetic volume of 2% lidocaine in ultrasound-guided axillary brachial plexus block.* The Journal of the American Society of Anesthesiologists, 2009. **111**(1): p. 25-29.
6. Spanos, S., et al., *Jet Injection of 1% buffered lidocaine versus topical ELA-Max for anesthesia before peripheral intravenous catheterization in children: a randomized controlled trial.* Pediatric emergency care, 2008. **24**(8): p. 511-515.
7. Levin, Y., E. Kochba, and R. Kenney, *Clinical evaluation of a novel microneedle device for intradermal delivery of an influenza vaccine: are all delivery methods the same?* Vaccine, 2014. **32**(34): p. 4249-4252.
8. Levin, Y., et al., *Intradermal vaccination using the novel microneedle device MicronJet600: Past, present, and future.* Human vaccines & immunotherapeutics, 2015. **11**(4): p. 991-997.
9. Carlsson, A.M., *Assessment of chronic pain. I. Aspects of the reliability and validity of the visual analogue scale.* Pain, 1983. **16**(1): p. 87-101.

The list of criteria for inclusion, non-inclusion of subjects in the study and exclusion of subjects from the study " Use of MicronJet600 hollow micro-needle injection system for lidocaine intradermal administration to ensure local anesthesia when inserting peripheral venous catheter"

Criteria for inclusion of subjects in the study:
1. The presence of a written informed consent of the subject to participate in the study;
2. Age 18-65 years;
3. Any gender;
4. The possibility of subject-friendly insertion of peripheral 18G catheter into the median cubital vein, no contraindications for this.

Criteria for non-inclusion of subjects in the study:
1. In women: pregnancy, breastfeeding;
2. The presence in the history of an allergic reaction to lidocaine;
3. The presence of a syndrome of fear of sharp objects;
4. The presence of concomitant pain syndrome;
5. The presence of any tissue damage in the area where the 18G peripheral catheter is inserted into the cubital vein.

Criteria for exclusion of subjects from the study:
1. Refusal of the subject from participation during the study.
