# Supplementary material for: Intradermal injection of lidocaine with a microneedle device to provide rapid local anaesthesia for peripheral intravenous cannulation: A randomised open-label placebo-controlled clinical trial
Source: PLoS One. 2022 Jan 31;17(1):e0261641. doi: 10.1371/journal.pone.0261641 (PMC8803196; doi:10.1371/journal.pone.0261641)
Supplement: S2 File — (PDF) [file pone.0261641.s005.pdf]

Федеральное государственное автономное образовательное учреждение высшего образования
Первый Московский государственный медицинский университет имени И.М. Сеченова
Министерство здравоохранения Российской Федерации
(Сеченовский Университет)

АННОТАЦИЯ

клинического исследования

на тему:

Использование микроиглы MicronJet600 для внутрикожного введения лидокаина с целью обеспечения местного обезболивания при установке периферического венозного катетера

Ответственный исследователь: доктор медицинских наук, профессор, заведующий научно-исследовательского отдела инновационной терапии ФГАОУ ВО Первый МГМУ им. И.М. Сеченова Минздрава России (Сеченовский Университет), врач отделения гепатологии клиники пропедевтики внутренних болезней, гастроэнтерологии и гепатологии им. В.Х, Василенко УКБ № 2, Павлов Чавдар Савов.

Координатор проекта: младший научный сотрудник отдела биомедицинской инженерии ФГАОУ ВО Первый МГМУ им. И.М. Сеченова Минздрава России (Сеченовский Университет), Ржевский Алексей Сергеевич.

1. Актуальность и новизна темы по литературным источникам и патентной документации.

Идеальная техника для обеспечения местной анестезии должна быть простой, эффективной, быстродействующей, рентабельной, вызывать минимальный дискомфорт/боль у пациента . Несмотря на то, что уже существует большое количество методов обеспечения местной анестезии, каждый из них имеет свои ограничения. Так, стандартные подкожные инъекций являются наиболее распространенным средством введения местных анестетиков и позволяют быстро достигнуть желаемого результата. Однако, подкожные иглы могут вызывать нежелательные для пациентов боль и состояние стресса, особенно у пациентов с синдромом боязни острых предметов [2]. В противовес, доступные гели и кремы на основе местных анестетиков обеспечивают безболезненное достижение анестезии, однако время между их нанесением на поверхность кожи и достижением обезболивающего эффекта составляет около часа, что существенно ограничивает их применимость [3]. В последние годы были так же разработаны, несколько минимально- и неинвазивных методик внутрикожного введения местных анестетиков, среди них: ионтофорез [4], ультразвук [5], “струйная инъекция” [6]. Однако, ввиду сложности и дороговизны конструкции необходимых приспособлений, данные методики не возымели больших - коммерческого успеха и широты практического применения.

Так, в планируемом исследовании мы изучим эффективность минимально-инвазивного и безболезненного внутрикожного введения местного анестетика, и как следствие достижения местной анестезии на примере установки периферического венозного катетера в кубитальную вену, с помощью коммерчески доступной, и зарегистрированная на территории Российской Федерации (ригистрационное удостоверение от 23 марта 2017 года, см. приложенный документ) микроиглы MicronJet600 (NanoPass Technologies Ltd., Израиль) на основе полых микроигл [7, 8]. Данная инъекционная система была разработана для безболезненного внутрикожного введения лекарственных средств, с прицелом на вакцинацию [8], В нашем же исследовании ожидается высокая эффективность применения данной системы для обеспечения местной анестезии при внутрикожном введении малых доз 2% раствора лидокаина.

2. Цель и задачи планируемого исследования.

Основная цель планируемого исследования – раскрыть потенциал безболезненного и эффективного применения полых микроигл для обеспечения местной анестезии, на примере внутрикожного введения малых доз лидокаина с помощью микроиглы MicronJet600.

Задачами исследования являются:

а) Оценить эффективность внутрикожного введения малых доз 2% раствора лидокаина с помощью MicronJet600, с целью снижения болезненности процедуры постановки периферического венозного катетера.

б) Установить возможные побочные явления внутрикожного введения лидокаина с помощью MicronJet600.

в) Разработать рекомендации по использованию MicronJet600 с целью снижения болезненности процедуры установки периферического венозного катетера.

3. Планируемый вид клинического исследования.

Проспективное рандомизированное поперечное плацебо-контролируемое двусторонне-слепое клиническое исследование в параллельных группах.

4. Объект исследования и планируемое количество наблюдений.

Пациенты клиники пропедевтики внутренних болезней, гастроэнтерологии и гепатологии им. В. Х. Василенко УКБ № 2. в количестве 80 человек.

5. Конкретные методики планируемого исследования и метод статистической обработки результатов.

Боль, испытываемая пациентами во время постановки катетера калибром 18G в кубитальную вену будет оценена по визуальной аналоговой шкале (“visual analog scale”) [9]. Полученные результаты будут статистически обработаны с помощью двухвыборочного t-теста, с коэффициентом достоверности установленным на уровне $p < 0.001$.

Дизайн клинического исследования представлен в виде таблицы:

	Группа1		Группа2	
	Левая рука	Правая рука	Левая рука	Правая рука
**Описание установки периферического венозного катетера**	Катетер калибра 18G будет установлен в кубитальную вену сразу после внутрикожного введения 0.1 мл 2% раствора лидокаина в области пункции.	Катетер калибра 18G будет установлен в кубитальную вену сразу после внутрикожного введения 0.1 мл физиологического раствора в области	Катетер калибра 18G будет установлен в кубитальную вену сразу после внутрикожного введения 0.1 мл 2% раствора	Катетер калибра 18G будет установлен в кубитальную вену без предвариельного введения лидокаина или физиологического раствора.

		пункции, в качестве плацебо.	лидокаина в области пункции.	
**Количество пациентов**	N=40		N=40	
**Проводимые исследования**	Боль, испытанная пациентами во время процедуры будет оценена ими по предоставленной 100-балльной визуальной аналоговой шкале.<br><br>В качестве дополнительного исследования, область пункции будет осмотрена на наличие припухлости, покраснения и кровоподтёков через час после использования MicronJet600 во всех случаях. Все жалобы, предъявленные пациентами, и непосредственно относящиеся к данному исследованию, будут фиксироваться. Так же, в случае введения лидокаина, будет оценена длительность потери кожной чувствительности (далее ДПКЧ) с помощью одноразовой мелкокалиберной иглы, калибром 27 G и длиной 13мм, за счёт её точечного контакта с кожей, то есть соприкосновения кончика иглы с поверхностью кожи, на расстоянии в 1, 2 и 3 см от места введения лидокаина. Оценка ДПКЧ будет произведена через 15, 30 и 45 минут после введения лидокаина. При оценке ДПКЧ, в каждом временном интервале будет использована новая одноразовая игла калибром 27 G и длиной 13мм. Количественно, потеря кожной чувствительности, то есть боль, испытуемая пациентами при описанном контакте поверхности кожи с иглой калибром 27 G и длиной 13мм, будет так же оценена с помощью 100-балльной визуальной аналоговой шкалы.			

Таким образом, ввиду того, что в форме письменного информированного согласия пациенты будут ознакомлены с основной концепцией исследования (использование MicronJet600 для снижения болезненности процедуры постановки периферического венозного катетера посредством внутрикожной инъекции анестетика), плацебо-контролируемое исследование с пациентами из первой группы позволит доказать отсутствие субъективности восприятия пациентами процедуры применения MicronJet600, и наличие реального эффекта снижения болезненности установки периферического венозного катетера после внутрикожного введения 2% раствора лидокаина. В свою очередь, исследование с пациентами из второй группы позволит оценить степень снижения болезненности процедуры установки периферического венозного катетера после внутрикожного введения лидокаина с помощью MicronJet600. в сравнении со стандартной техникой установки периферического венозного катетера. При проведении исследования, в случае с каждым пациентом, манипуляции будут проводиться в начале с правой, а затем с левой рукой.

Рандомизированное распределение пациентов по двум исследуемым

группам будет произведено с помощью метода последовательных номеров. Так, каждому пациенту будет присвоен номер, который будет являться случайным числом из таблицы случайных чисел. Далее, пациенты с присвоенным четным номером будут отнесены к первой исследуемой группе, а пациенты с нечетным номером ко второй исследуемой группе.

Все манипуляции, относящиеся к настоящему исследованию, будут проведены в соответствии с Хельсинской Декларацией Всемирной Медицинской Ассоциации и принципами надлежащей клинической практики.

6. Предполагаемый результат исследования.

Будет отражена практическая эффективность предпункционного обезболивания посредством внутрикожного введения малых доз 2% раствора лидокаина с помощью микроиглы MicronJet600, и разработаны рекомендации для наиболее успешного проведения данной процедуры.

7. База проведения научного исследования.

ФГАОУ ВО Первый МГМУ им. И.М. Сеченова Минздрава России (Сеченовский Университет), Клиника пропедевтики внутренних болезней, гастроэнтерологии и гепатологии им. В. Х. Василенко УКБ № 2.

8. Поддержка со стороны организаций.

Для данного исследования, микроиглы MicronJet600 будут любезно предоставлены компанией NanoPass Technologies Ltd., Израиль.

Ответственный исследователь: Павлов Ч.С.

Координатор проекта: Ржевский А.С.

9. Список литературы

1. Houck, C.S. and N.F. Sethna, *Transdermal analgesia with local anesthetics in children: review, update and future directions.* Expert review of neurotherapeutics, 2005. **5**(5): p. 625-634.
2. Thurgate, C. and S. Heppell, *Needle phobia–changing venepuncture practice in ambulatory care.* Paediatric nursing, 2005. **17**(9): p. 15-18.
3. Zempsky, W.T., *Pharmacologic approaches for reducing venous access pain in children.* Pediatrics, 2008. **122**(Supplement 3): p. S140-S153.
4. Zempsky, W.T., et al., *Evaluation of a low-dose lidocaine Iontophoresis system for topical anesthesia in adults and children: A randomized, controlled trial.* Clinical therapeutics, 2004. **26**(7): p. 1110-1119.
5. O'Donnell, B.D. and G.J.A.T.J.o.t.A.S.o.A. Iohom, *An estimation of the minimum effective anesthetic volume of 2% lidocaine in ultrasound-guided axillary brachial plexus block.* 2009. **111**(1): p. 25-29.
6. Spanos, S., et al., *Jet Injection of 1% buffered lidocaine versus topical ELA-Max for anesthesia before peripheral intravenous catheterization in children: a randomized controlled trial.* Pediatric emergency care, 2008. **24**(8): p. 511-515.
7. Levin, Y., E. Kochba, and R. Kenney, *Clinical evaluation of a novel microneedle device for intradermal delivery of an influenza vaccine: are all delivery methods the same?* Vaccine, 2014. **32**(34): p. 4249-4252.
8. Levin, Y., et al., *Intradermal vaccination using the novel microneedle device MicronJet600: Past, present, and future.* Human vaccines & immunotherapeutics, 2015. **11**(4): p. 991-997.
9. Carlsson, A.M., *Assessment of chronic pain. I. Aspects of the reliability and validity of the visual analogue scale.* Pain, 1983. **16**(1): p. 87-101.

Перечень критериев включения, невключения пациентов в исследование и исключения пациентов из исследования «Использование микроиглы MicronJet600, на основе полых микроигл, для внутрикожного введения лидокаина с целью обеспечения местного обезболивания при установке периферического венозного катетера».

Критерии включения пациентов в исследование:
1. Наличие письменного информированного согласия пациента на участие в исследовании;
2. Возраст 18-65 лет;
3. Любой пол;
4. Возможность безопасной для пациента постановки периферического катетера размером 18G в кубитальную вену, отсутствие противопоказаний для этого.

Критерии невключения пациентов в исследование:
1. У женщин: беременность, кормление грудью;
2. Присутствие в анамнезе аллергической реакции на лидокаин;
3. Присутствие синдрома боязни острых предметов;
4. Наличие сопутствующего болевого синдрома;
5. Присутствие каких-либо повреждений тканей в области постановки периферического катетера 18G в кубитальную вену.

Критерии исключения пациентов из исследования:
1. Отказ пациента от дальнейшего участия в исследовании.
