# Supplementary material for: Intradermal injection of lidocaine with a microneedle device to provide rapid local anaesthesia for peripheral intravenous cannulation: A randomised open-label placebo-controlled clinical trial
Source: PLoS One. 2022 Jan 31;17(1):e0261641. doi: 10.1371/journal.pone.0261641 (PMC8803196; doi:10.1371/journal.pone.0261641)
Supplement: Attachment — Submitted filename: Response to Reviewers.docx [file pone.0261641.s006.docx]

Reviewer #1: The authors conducted a (partly) double-blinded three arm placebo-controlled clinical trial to compare the Intradermal injection of lidocaine with a placebo (100μL of saline) as well as doing nothing to provide rapid local anaesthesia for peripheral intravenous cannulation with respect to pain after cannulation. The comparison with lidocaine was done with intraindividual comparisons. They concluded, that the described MicronJet600 technique was safe and effective for providing rapid local anesthesia. The technique is also considered promising for local anesthesia in such procedures as blood gas sampling and minor surgical interventions such as excision of small skin lesions, or surgical sutures removal.

There are major points from the methods point of view which conflict the validity, but may be possibly addressed in a review.

The authors are encouraged to include an individual patient data file which may be anonymized, but let the reviewer and the reader follow the arguments.

Second, I welcome very much the effort to make the presentation consistent with the CONSORT guideline. My impression is, that this is mostly done successful. However, every point of the CONSORT like 3b,6b,7b, 11b, 14b, 17b and 20 should be addressed, at least with the statement like “no changes to the protocol are made”.

The missing line numbers influence the style of my comments:


The general comments are:
1. Restructuring along the CONSORT checklist recommendation is necessary. Some parts of the introduction belong to the Method section (treatments). Most of the study designs section is in-/exclusion criteria (section setting) as well as treatment (section interventions) which does not match to study design. A separate figure explaining the study design is helpful.

Response: Details which may belong to the Method section have been removed from the last paragraph of the introduction. Necessary amendments to the study design section have been made. As a separate figures explaining the study design we have provided Fig 2 ; Fig 3 “Consolidated standards for reporting of trials diagram” also supports the explanation. Response: A required changes in the CONSORT checklist have been made.

Please give details / differences between applied treatments to illustrated mantainance to blinding.

Response: Necessary details have been provided.

2. The conclusion is not supported by the data. A reformulation along the primary hypothesis is necessary.

Response: We have formulated primary hypothesis in the introduction. Relevant data has been added to the conclusion.

3. A limitation section is missing in the discussion section.

Response: Relevant information has been added as a separate paragraph in the Discussion section.

4. A statement about the ethical consideration, i.e. conduct in a group of healthy volunteers, may be worth to include in the discussion.

Response: Relevant information has been added at the beginning of the first paragraph of the Discussion section.

5. The number of patients (including diseases) and healthy volunteers should be given.

Response: The information has been added to an Excel file which has been uploaded to the ResearchRegistry.

6. The intraindividual comparisons are confounded with the application side. This effect should by addressed in the limitation section “as unclear effect on the study result” and has implication on the conclusion.

Response: Relevant information was added at the end of the last paragraph of the Discussion section.

7. The study is only blinded for the group1 comparison. Group2 is at most observer blinded. Effectiveness of “Blinding” is difficult to assess, as the allocation is determined by side and thus only the allocation to the type of the control group might be unclear. This should be made clear; the study design classification should be “open label” instead of blinded. The authors are welcome to describe the efforts to implement blinding.

Response: In case of Group1, as well as in case of Group2, Observer2 who examined subjects after the cannulations was not aware about the details of the intervention (which arm received which type of treatment). Also, the nurse implementing the interventions was not aware about the details of the intervention (which arm received which type of treatment). Yes, in case of Group2, as well as in Group1, the subjects could see an application of MicronJet600. However, the patients in both groups did not know what kind of drug was injected in which arm.

The blinding issue has been added as one of the limitations in the discussion section: “Also blinding could has been performed more rigorously although it is difficult to blind (disguise) the use of the MicronJet600 device; the intraindividual comparisons are confounded with the application side, which has an unclear effect on the study result; since there is no evidence regarding the difference of pain sensitivity between arms in population, arms were not randomized.”

8, The abstract is not structured according to CONSORT recommendation.

Response: The abstract has been structured into the sections: Background, Methods, Findings, Conclusion.

9. Definition of the primary endpoint variable is missing in the abstract as well as the method section. Please give the time of evaluation when defining the primary endpoint variable as well.

Response: Primary endpoint variable has been characterised in the “Intervention” section.

10. Characterization of secondary variables is missing.

Response: Secondary endpoint variable has been characterised in the “Intervention” section.

11. At the end of the “Introduction” a clearly specified primary hypotheses (which can be answered by a suitable statistical test) is missing.

Response: A primary hypothesis has been specified at the beginning of the last paragraph in the introduction section. It contains a “pain score” quantitative variable, and therefore can be answered by a suitable statistical test.

12. The text following “The results of this ..” should be deleted, while no data are presented or published.

Response: Relevant data has been uploaded to the ResearchRegistry with an Excel file.

13. Give the location of the clinic in the setting section.

Response: Location of the clinic has been mentioned in the Materials and methods section.

14. Give the initials of the observers.

Response: The initials of the observers have been given.

15. Please give explanation, what is gained from the second control group. Although the saline group can be followed the second control group is difficult to understand. Note, the comparison for the MicronJet600 technique (control group1 vs control group2) is not indicated as a primary research question.

Response: In the Introduction section we indicate that the conducted study is placebo controlled. To demonstrate that the placebo (saline) had no effect, statistically we compared pain score experienced by subjects from group1 due to cannulation performed after placebo injection with the pain score experienced by subjects from group2 to cannulation performed without any pretreatment. This was mentioned in the discussion section.

16. The comparison within group2 (lido vs no pretreatment) does not answer the question about MicronJet600 use, as the treatment is confounded with the MicronJet600 use.

Response: This has been mentioned as one of the limitations of the study.

17. The statistical analysis model may be inadequate, depending on the primary research question/hypothesis. In particular with the comparisons to the lido pretreatment more data are available, than used for the particular analysis. A suitable linear mixed model might be helpful to address intra-patient variability as well as all treatments. Linear contrast help to answer specific questions while protecting for type one error inflation.

18. Details of the repeated measures ANOVA model should be given. I do not see two between factors here. Linear mixed effects model with a clear statement about within factors, random factors and covariance structure assumption is necessary (see comment 17).

Responses for 17, 18: We have used linear mixed effects model with unstructured variance-covariance matrices. Detailed description of model specifications and estimation have been included to “Methods” section. The only assumption on conditional normal distribution of residuals have been visually checked using QQ-plots (not included to the manuscript). Heterogeneity of variance have been addressed using robust variance-covariance estimators.

19. Sample size calculation is difficult to understand, which the primary endpoint is not referred to, a reference of the numbers is not given and the clinical relevance of the effects is not clear, the link to a hypothesis reflecting the design is not mentioned.

Response: We have included more clear description of sample size calculation. But of course the limitation of the study is discrepancy between power analysis model and model implemented in the study. We have included this issue to “Discussion” section.

20. P-Values should be given by at least 3 decimal digits.

Response: P-values have been given by 3 decimal digits.

21. Classification of VAS scale seem to be arbitrary.

Response: A provided classification of VAS scale has been supported by ref. [20]. Considering that VAS-scale is a pretty common tool for measuring pain score in clinical trials, this should be sufficient for readers to understand a specificity of VAS.

Reviewer #2: Thank you for the opportunity to review your manuscript. This study concerns patient’s perception of IV cannulations and evaluates a device for ensuring immediate pain relief as an alternative to topically applied local anesthetics that requires a pause in the procedure. The design is described as a randomized double-blinded placebo controlled clinical trial and according to the study synopsis the study also follows GCP guidelines.

However, the study, as described both in researchregistry.com, the study synopsis and in the manuscript, has substantial flaws in its design. The randomization has not been implemented to optimize the study trustworthiness and the blinding procedures are insufficient. 

Introduction:
How do you substantiate the claim that MicronJet600 is “the foremost device” of the commercially available?

Response: The word “foremost” has been removed from the manuscript.

The last paragraph would benefit by removing the comparison with the previous device and instead focus on presenting the primary and secondary objectives of the study, preferentially as testable hypothesis.

Response: Excess details regarding the previous device are removed from the text. Primary and secondary objectives have been described in the “Study objectives” section.

Methods
Who prepared the MicroJet600 devices before use, i.e. added saline or 2% lidocaine? Observer 1? Was it the same research nurse that inserted the 18G needles? Why was not the right vs left arm also randomized to avoid potential systematic technical issues with the IV cannulations and also blind the observers? Was the the order between right and left arm cannulations identical between all subjects?

Response: Yes, Obsever1 prepared the MicronJet600 devices before use. No, Observer1 and research nurse are different people. Since there is no evidence in literature that pain sensitivity may vary between arms in population, we decided that this is not essential to randomize the arms. We have added this point to the limitations of our study. The Observer2 who examined patients after the cannulations was blinded, the observer was not aware about the details of the trial (which arm receives which treatment). Yes, the order between right and left arm cannulations was identical between all subjects.

The randomization appears to be into groups that received lidocaine (left arm) – saline (right arm), or lidocaine (left arm) – no injection (right arm). Thus, according to the protocol that was made public before the first patient was included, both the research nurse and observers potentially knew which arm that in all cases had been injected with lidocaine. This is hardly a blinded protocol. Furthermore, from the patient’s point of view the first group might have been considered blinded provided that the protocol had stipulated a randomization of which arm that received saline or lidocaine. But this was not the case according to the protocol.

Response: The Observer1 prepared the syringes for the nurse without indicating which syringe contains which drug (lidocaine or placebo. The nurse didn’t know which syringe contains which drug, and visually the syringe containing lidocaine is the same as the syringe containing the placebo. The nurse was given an information which syringe she must use for which arm. The patients did not know which arm received which drug – placebo or lidocaine, even in case of Group2 they were not aware whether the arm receives lidocaine or saline.

The blinding issue has been indicated as one of the limitations of this study in the “Discussion” section

Statistics
How did you test the distribution of the data, i.e. whether parametric tests are valid and mean +-SD is a valid descriptor?

Response: In the revised version of the manuscript, standard deviations have been switched to confidence intervals.

Results
The results section can be substantially shorter and would benefit from being presented in line with the hypotheses that are tested. 
Response: The results section has been shortened and re-written in line with the hypothesis.

Table 1: typo with a , instead of a .

Response: We apologise but we haven’t find a mistake in Table 1.

Protocol considerations
The procedure for blinding of the patient and of the observers are flawed. The protocol that was made public before the start of the inclusion of patients states that all injections with active substance are made in the left arm, and placebo or no injection are always done in the right arm. Also Observer 1, who prepared the devices for injection, are involved in evaluations of the patients.

Response: There is no evidence that pain sensitivity differs between arms in population. Observer1 wasn’t involved at the stage of evaluation of the primary endpoint variable (pain score of the cannulations). Observer1 got involved in evaluation of the patients only after measurement of cannulation-induced pain, at the stage of measuring the distance and duration of skin numbness.

The amount of pain inflicted on the patients by the injection of placebo or active substance, compared to the pain inflicted by the insertion of the 18 G needle would by itself have been an interesting comparison.
Response: A measurement of pain score due to intradermal injection of active substance had been performed in the previous clinical trial, where MicronJet device was used, and described in the discussion section.

Discussion
Please remove unpublished data from discussion. It does not substantiate the publication. Again, please state aims and hypothesis in introduction.

Response: The aims and hypothesis have been stated in the introduction.

Data obtained in the previous clinical trial, where MicronJet device was used, supplement data from the current study. We would like to keep it, if this is not a problem.

Conclusion
Should be directly in line with the stated objectives. Please avoid other assumptions in the conclusion.

Response: The conclusion has been rewritten in line with the stated objectives

Financial disclosure
The text in the manuscript does not match the text in the additional information.

Response: Information regarding financial disclosure has been presented in accordance with the journal’s requirements.

Data availability
The datapoints has not been made available in this version. Please see the last sentence:

Response: Data has been uploaded as an Excel file to a ResearchRegistry public repository (registration number: researchregistry4662, principal investigator: Chavdar Pavlov, date of registration: 29 January 2019, URL: https://www.researchregistry.com/browse-the-registry#home/registrationdetails/5c4d811ac413740862094f0f/)

”The PLOS Data policy requires authors to make all data underlying the findings described in their manuscript fully available without restriction, with rare exception (please refer to the Data Availability Statement in the manuscript PDF file). The data should be provided as part of the manuscript or its supporting information, or deposited to a public repository. For example, in addition to summary statistics, the data points behind means, medians and variance measures should be available.”

Reviewer #3: Intradermal injection of lidocaine with a microneedle device to provide rapid local anaesthesia for peripheral intravenous cannulation: A randomised double-blind placebo-controlled clinical trial

Nice study, very Practical among the pediatric population and can be used in adult as well.

This study is done well with good statistical analysis.

In our institute we start using the J-tip and you can check it on www.jtip.com

It's Needle-free injecting System for subcutaneous administration of Novocaine

We found some benefit from using Compared to the EMLA cream

In the mean while we are generating a study very close to your study.

Response: We are thankful for such a positive opinion on our study, and are very happy that this area of research attracts significant attention.
