# Supplementary material for: Intradermal injection of lidocaine with a microneedle device to provide rapid local anaesthesia for peripheral intravenous cannulation: A randomised open-label placebo-controlled clinical trial
Source: PLoS One. 2022 Jan 31;17(1):e0261641. doi: 10.1371/journal.pone.0261641 (PMC8803196; doi:10.1371/journal.pone.0261641)
Supplement: Attachment — Submitted filename: Response to Reviewers2, 04.03.2021.docx [file pone.0261641.s007.docx]

Reviewer #2: Thank you for addressing the comments and vastly improving the manuscript. Now it is easy to follow your design and experimental setup.

A major issue remains. In this study, all patients according to the protocol received the active substance in the right arm and the left arm was always control. The randomization was to either receive saline injected in the left arm or not to receive an injection of saline. This was known to the investigators that made the measurements.

The protocol stating this procedure, the active substance in the right arm and saline/no-treatment in the left arm, was published on the web. Thus it cannot be ruled out that also the test subject knew which side that received the active substance. Accordingly, the study can not be said to be blinded. This needs to be changed throughout the title, abstract and manuscript.

Response: Yes, the blindness could has been performed more rigorously. In the title, abstract and manuscript “double-blinded” was changed to “open-label”.

The conclusion should be based on data from this study and answer the aim of the study. Thus the first sentence in the conclusion in the abstract is relevant but not the second sentence. I suggest that you delete it.

Response: Done.

The conclusion in the main text is currently a repetition of results and also a sales pitch for more uses of the device. It needs to be revised.

Response: An excess information regarding the results of the study was removed from the “Conclusions” section. However, we can not entirely rewrite the conclusions because conclusions are always drawn from results.

Reviewer #4: The authors performed a double-blinded randomized trial to assess the efficacy and safey of a microneedling device to provide rapid anesthesia for the insertion of a peripheral intravenous catheter. 100 health volunteers were randomized into two groups: one with microneedle with lido 2% and saline in contralateral arm, second group one with microneedle with lido 2% and no injection in the contralateral arm. The subjects served as there own control. An 18G cannula was inserted in both arms. Intradermal injection with lido 2% reduced pain significantly and subjects preferred lido. It was concluded that injection with microjet600 was safe and effective option for rapid local anesthesia for peripheral iv cannulation.

Control group that compared a to a regular intradermal injection is not included. So it is not clear whether the injection with the micronjet600 is necessary to achieve the effect.
Would a normal intradermal needle would provide the same result?

Response: Lidocaine injection with a normal hypodermic needle would probably have similar efficacy in terms of anaesthesia. However, because injection with a normal hypodermic needle is painful and highly-invasive, it is commonly not used in clinical practice prior to intravenous cannulation. Thus, minimally invasive and none-invasive approaches for intradermal delivery of anaesthetics have been actively investigated, please see a systematic review by Bond M, Crathorne L, Peters J, et al. 2015 (the first reference in the article). In most of the clinical trials investigating such minimally invasive or none-invasive approaches, control group with a regular intradermal injection was not included. As an example of such studies, please see Spanos S, Booth R, Koenig H, et al. 2008 (reference 7 in the article) and Lysakowski C, Dumont L, Tramèr MR, Tassonyi E 2003 (reference 23 in the article).

Was the person that applied the lido 2%/saline with the micronjet blinded?

Response: Yes, the nurse who applied the lido 2%/saline with the MicronJet600 was blinded.

P6, sample size ◊ calculation of the sample size is unclear. Wat is meant by effect size 1 with and SD of 2.2? Is one point on the VAS score meant here?

Response: The following has been added to the text of the article in the “Sample Size” section, marked with yellow:

“A sample size of 40 subjects per group was calculated to detect an effect size (expected difference on the VAS score between two time points at the specific distance) 1 with standard deviation of the effect at 2.2 using a paired t-test with 80% power and 5% type I error rate assuming two-sided significance testing procedure. At the same time, additional 22 subjects (102 subjects in total) were enrolled in order to overcome dropout scores.”

P9, adverse events ◊ there was not powered for adverse events, question arises whether safety can be really assessed based on these data. However, it is not expected that intradermal injections cannot be considered safe, as local anesthesia with lidocaine is common practice.

Response: The limitation has been added to the text of the article.

P9, first paragraph of discussion: the results do not have to be repeated. Suffices to state main findings here.

Response: The information on the results, presented in the first paragraph of the “Discussion” section, is coherent with the discussion of these results. We greatly apolagise but in our opinion, this information can not be just simply removed without compromising the overall meaning of the discussion.

Limitations:
No control with normal needle. The notion that the use of MicronJet600 is speculative

Response: We agree that the study was not powered for adverse events, we did not perform statistical testing procedures on these data. The limitation has been added to the text of the article.

Reviewer #5: The authors have responded to most of the issues that Reviewer 1 listed about the statistical aspects of the paper. There are a few remaining issues:
1. The randomization procedure: complete, permuted blocks, etc. (see Rosenberger and Lachin, Randomization in Clinical Trials, 2016, Wiley for details). Excel is NOT a randomization procedure.

Response: The following has been added to the text of the article in the “Participants” section:

Response: Simple randomization was preformed to allocate subjects into 2 groups using Microsoft Excel random number generator.

2. Sample size is still unclear. What is a treatment effect of 1? Does this mean a change in mean VAS of 1? Be more specific. Where is the standard deviation of 2.2 from? You need to reference a pilot study or the literature.

Response: The following has been added to the text of the article in the Sample Size section, marked with yellow:

“A sample size of 40 subjects per group was calculated to detect an effect size (expected difference on the VAS score between two time points at the specific distance) 1 with standard deviation of the effect at 2.2 using a paired t-test with 80% power and 5% type I error rate assuming two-sided significance testing procedure. At the same time, additional 22 subjects (102 subjects in total) were enrolled in order to overcome dropout scores.”

3. There are voluminous missing and incorrect definite and indefinite articles.

Response: The manuscript has been revised and necessary corrections have been made. However, we would be happy if the Reviewer #5 specify the incorrect definite and indefinite articles.
