# Supplementary material for: Intradermal injection of lidocaine with a microneedle device to provide rapid local anaesthesia for peripheral intravenous cannulation: A randomised open-label placebo-controlled clinical trial
Source: PLoS One. 2022 Jan 31;17(1):e0261641. doi: 10.1371/journal.pone.0261641 (PMC8803196; doi:10.1371/journal.pone.0261641)
Supplement: Attachment — Submitted filename: Response to reviewers3, 08.05.2021.docx [file pone.0261641.s008.docx]

Reviewer #2: Thank you for the adjustments to the manuscript. It addresses most of my concerns.

However, the present version of the submission still have issues that needs to be addressed.

Comment: Please update the abstract in the submission system to be identical with the abstract in the manuscript.

Response: Done

Comment: The study cited on page 3 was registered 2007 and has not yet been published. I would suggest that you remove the last sentence in the paragraph completely as the claimed support for the results cannot be verified.

Response: Done

Comment: The language needs to be edited by English editing service.

Response: Edited

Examples:
Comment: "Consequently, the average pain scores for cannulation with no pretreatment and cannulation with the preliminary intradermal injection of the placebo were comparable in both studies – the current study and the study by Lysakowski et al.". Please rewrite without the –

Response: Done

Comment: "Thus, the average 100-poin VAS pain scores versus 10-poin NVS were: 39.7 and 3.9, and 41.5 and 4.2 for cannulation". Should be point instead of poin.

Response: Done

Comment: "It is anticipated that the technique is appropriate for a middle-term small surgical procedures such as excision of small skin lesions, or surgical skin staples or sutures removal [24], nevus removal. " Should be rephrased into correct english.

Response: Done

Comment: Table 1
An accidential , instead of .

Response: Corrected

Comment: Citing of references needs to be consistent and according to PlsOne standards. Eg, in the same paragraph the same paper is referenced differently:
...in comparison with the study by Lysakowski, Dumont, Tramer and Tasonyi. (500 μL) [23]
- The last author misspelled
...the study by Lysakowski et al. [23]

Response: Done

Comment: Including own unpublished data as in the last paragraph in the discussion (page 10) is still not appropriate as the results cannot be verified against methods used, nor have they been peer-reviewed. If the results holds for dissemination they should be published within in the present or in a separate study.

Response: The paragraph has been removed

Comment: Conclusion
I expect the conclusion to be brief and directly related to the aims of the study. It would benefit by being substantially shorter and not be a repetition of the results.

Response: Done

Reviewer #4: This is the revised version of the manuscript “Intradermal injection of lidocaine with a microneedle device to provide rapid local anaesthesia for peripheral intravenous cannulation: A randomised open-label placebocontrolled clinical trial” by Rzhevskiy and co-authors.
The manuscript has improved and only minor issues remain.


Comment: Discussion

The first paragraph of the discussion should state the main findings, a short summary, without repeating the results section.

Response: Done

Comment: Figures
Resolution of the figure 2 and 3 are not optima land should be improved

Response: Improved

Comment: Figure 2
Layout figure 2 is not optimal and can be improved

Response: Improved

Comment: Figure 5

Is figure correct? In this figure the total of the bars per group should be 100%, this seems to be the case in the no pretreatment group and placebo group, but not in the 2% lido group: when the total of the bars is more than 100%

Response: Yes, the figure is correct. In this figure, the total of the bars per group should not be 100%. For better understanding, such category as VAS-score ⩽ 20 includes all cases from 0 to 20, therefore this category at the same time involves such categories as VAS-score ⩽ 10 and VAS-score=0.

Reviewer #5: (No Response)
